# Short-term effects on physical activity level with web-based self-management support in people with COPD: a randomised controlled trial

**DOI:** 10.1038/s41533-024-00394-7

**Published:** 2024-10-24

**Authors:** Tobias Stenlund, Åsa Karlsson, Per Liv, André Nyberg, Karin Wadell

**Affiliations:** 1 Department of Community Medicine and Rehabilitation, Section of Physiotherapy, Umeå University, Umeå, Sweden; 2 Department of Public Health and Clinical Medicine, Section of Sustainable Health, Umeå University, Umeå, Sweden

**Keywords:** Rehabilitation, Patient education

## Abstract

We aimed to evaluate short-term effects of a web-based self-management support on objectively measured physical activity (PA) compared to usual care in people with chronic obstructive pulmonary disease (COPD). We conducted a pragmatic randomised controlled trial including people with stable COPD within primary healthcare. Participants were randomised to intervention group, IG (access to the COPD Web, an interactive website to support self-management with focus on PA), or to control group, CG (usual care). Primary outcome at 3 months was change in accelerometry-measured daily steps analysed with ANCOVA, and secondary outcomes were self-reported PA, disease-related symptoms, and quality of life. Missing data in intention-to-treat (ITT) analyses were multiply imputed. One hundred and forty-six participants (*n* = 73/group), mean (SD) age 69.5 (6.7) years, FEV_1pred_ 60.7 (19.1)% were included. The ITT analysis showed no significant difference in steps between the groups: 1295 steps (95% CI: [−365, 2955], *p* = 0.12), while the complete case analysis (*n* = 98) revealed a significant difference of 1492 steps (95% CI: [374, 2609], *p* = 0.01) in favour of IG. A significant increase in self-reported PA was seen in IG in both the ITT and complete case analysis. In summary, access to the COPD Web was insufficient to increase short-term PA level compared to usual care. However, among participants with complete step data, a clinically relevant effect on daily steps exceeding the minimal important difference was observed, partly explained by higher baseline PA than among dropouts. This indicates that access to the COPD Web may increase PA levels for some people with COPD.

## Introduction

Decreased physical activity (PA) has been shown at an early stage of disease progression in people with chronic obstructive pulmonary disease (COPD)^1^. Lower number of steps per day independently predict future exacerbations and COPD-related hospitalisations^2^ and those who are more physically active have, independent of lung function, lower healthcare utilisation, lower risk of hospitalisation^3,4^ and death^5^.

Self-management support, including strategies for increased PA level, is a vital component of the management of COPD^6^. For instance, an increase of 600 steps per day has been found to be associated with a lower risk of hospitalisations^7^. Self-management interventions are directed towards behavioural change, which is particularly important when aiming to increase PA level^8^. The interventions are often personalised, structured and multicomponent^9^, and can, in addition to PA, target symptom management, smoking cessation and dietary intake^10^. They aim to empower the individual through increased knowledge, skills, and confidence in successfully managing the disease^6^. Studies have shown that people with COPD who use self-management strategies have improved health-related quality of life (HRQOL), decreased risk for respiratory-related hospital admissions, less depressive symptoms, and improved exercise capacity^10^. However, access to and knowledge about self-management strategies for people with COPD are limited in primary healthcare^11,12^.

Electronic health (eHealth), i.e., the use of information and communication technologies in health services, has emerged as a promising strategy to improve the limited access to evidence-based treatment in people with COPD^13,14^. People with COPD, as well as healthcare professionals (HCPs), have voiced that eHealth interventions can improve healthcare delivery and provide COPD self-management support^15^. Whether self-management support provided through a web-based platform or a smartphone application in combination with a pedometer could be used to promote PA needs further investigation as previous randomised controlled trials (RCTs) have shown both positive^16–19^ as well as no effects^20^ on PA levels. We have previously, in co-creation with people with COPD, their relatives, and HCPs, developed the COPD Web, to support people with COPD in their self-management strategies and to facilitate the provision of self-management support from the HCPs^21^. The COPD Web has been described as a suitable eHealth tool to provide self-management support^22^, and a pilot study showed that access to the COPD Web for 3 months increased self-reported PA levels, improved COPD-specific knowledge, and altered self-management strategies^23^. The primary aim of the present study was to evaluate the short-term effects of the use of the COPD Web on objectively measured PA level compared to usual care in people with COPD in primary healthcare. A secondary aim was to evaluate the short-term effects on self-reported PA level, dyspnoea, COPD-related symptoms, and HRQOL compared to usual care. We hypothesised that people who used the COPD Web as a strategy to change PA behaviour would increase their PA levels to a larger extent than people given usual care.

## Materials and methods

### Study design and participants

We conducted a pragmatic, parallel-group RCT in a primary healthcare context with a 3- and 12-month follow-up, of which the present study will report on the 3-month results. The trial was registered at ClinicalTrials.gov (NCT03746873) and the trial protocol was published^24^. Ethical approval was received from the Regional Ethical Review Board in Umeå, Sweden (Dnr: 2018-274-31, 2019-05572). All participants gave written informed consent to participate in the study. The study was reported following the CONSORT statement for RCTs and the extension for pragmatic trials^25^.

Participants were recruited through HCPs at 25 primary healthcare units situated in urban and rural areas in six different County Councils in Sweden and via advertisements in newspapers and Facebook during the period of November 2018 to March 2021. The last follow-up assessments were finalised in June 2022. During the trial, the Covid-19 pandemic caused restrictions from March 2020 to February 2022. Participants with stable COPD^26^ who made a regular visit to any of the involved primary healthcare units due to their COPD or those who contacted the research group due to advertisement were eligible for inclusion if they (1) could read and understand Swedish, (2) had a smartphone, tablet or computer with access to internet, (3) did not have dementia or other psychiatric condition that may prevent understanding of the intervention, (4) did not have severe comorbidity that could be considered as the contributing factor for limitation in PA, and (5) did not already use the COPD Web^24^. Results from participants’ latest pulmonary function test were used to verify the COPD diagnosis. After verbal agreement, an informed consent form, questionnaires, and an accelerometer for baseline assessment were sent to the participants.

### Randomisation and blinding

Participants were randomly allocated in a 1:1 allocation ratio to usual care with or without access to the COPD Web, after completion of the baseline assessment. A colleague, otherwise not involved in the study, performed the allocation using a computer-generated randomisation list and the results were stored in sealed envelopes. The randomisation was revealed for the researcher when the sealed envelope next in order was opened and the letter containing the allocation was sent to the participant. Blinding of participants and outcome assessors was not applicable since data was either self-reported or objectively measured by an accelerometer.

### Intervention

**Fig. 1:**
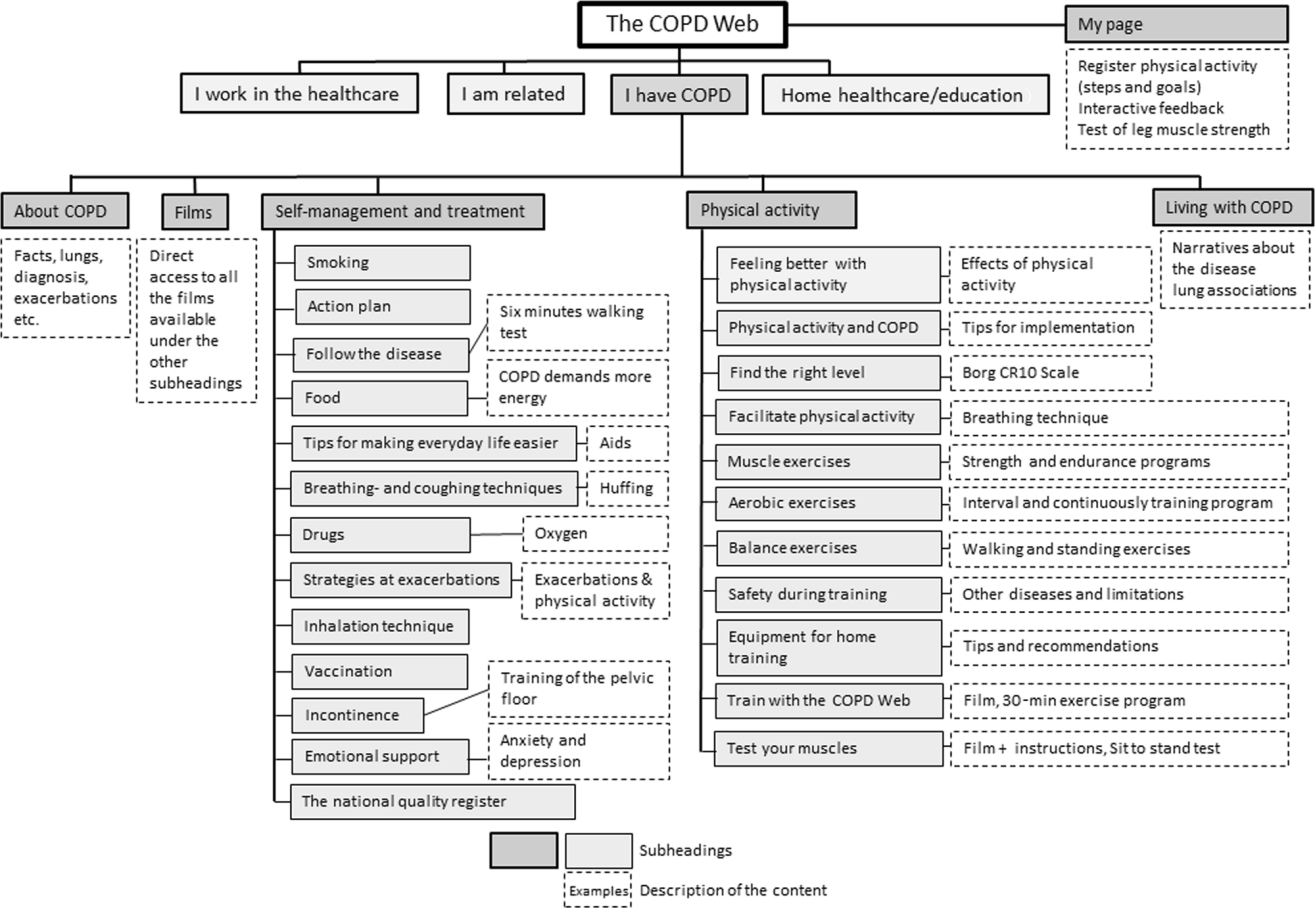
A website map of the COPD Web showing the section ‘I have COPD’^24^. Reprinted under the terms of the Creative Commons CC BY 4.0 license.

The intervention is previously described in detail^24^. In brief, the COPD Web aims to improve and support self-management of the disease with a special focus on PA. The COPD Web includes informative texts, pictures, films, and interactive components (i.e., step registration with automatised feedback), which aligns with the guidelines for COPD care developed by the National Board of Health and Welfare in Sweden^27^. An overview of the content is shown in Fig. 1. The intervention was designed to minimise the efforts required from HCPs and researchers. The participants were introduced to the COPD Web by a letter with written instructions on how to create an account. In addition, an instruction movie with standardised information was available on the website. They were also provided with a pedometer, a leaflet targeting the importance of PA, and information about when they would be contacted for the follow-up assessment. The participants in the intervention group were also contacted once by one of the researchers during the first week to reduce possible user problems. Thereafter, the participants received predetermined prompts via email and SMS, including targeted information, referral links to the COPD Web, and encouragement to register counted steps. The prompts were delivered once a week for the following 3 months. No other contacts between the participants and the researchers or the HCPS were included in the intervention. During the RCT, the COPD Web was secured with login and only minor adjustments, e.g., research news or new links, were added.

### Control group

All participants received usual care^24^. Similar to the intervention group, the control group received a pedometer with instructions and a leaflet targeting the importance of PA, but no other contacts besides the follow-up assessment. During the study, the participants were free to participate in other interventions in the community or at their primary healthcare unit.

### Outcomes

Full details on outcomes and assessment procedures are available in the study protocol^24^. Concisely, the primary outcome was the difference in PA level, measured as steps per day, between intervention and control groups at 3 months. The PA level was objectively measured for seven consecutive days using an accelerometer (DynaPort, McRoberts BV, the Netherlands). According to recommendations, only measurements with ≥4 valid weekdays were included, i.e., weekends were excluded, as well as weekdays with <8 h of daytime accelerometer wearing time^28^. Secondary outcomes were self-reported PA level measured by indicator questions from the National Board of Health and Welfare in Sweden^29^, dyspnoea measured by modified Medical Research Council dyspnoea scale (mMRC)^30^, HRQOL measured by the self-administered Chronic Respiratory Questionnaire (CRQ-SA)^31^, COPD-related symptoms measured by the COPD Assessment Test (CAT)^32^, and self-reported healthcare contacts.

### Sample size

The trial was designed to detect a mean difference of 1131 steps, which has previously been determined as a minimal important difference in people with COPD^7^. Demeyer et al.^7^ reported a standard deviation in PA level of 2193 steps after 3 months of Pulmonary Rehabilitation (PR) in a sample of people with COPD. Assuming the same standard deviation in the present trial and a dropout rate of 20% in both groups, 144 participants would be required to reach a power of 80% for detecting a group difference of 1131 steps when using a two-tailed independent sample t-test.

### Statistical methods

Descriptive statistics was calculated by group. Further, a dropout analysis comparing participant lost to follow-up with the full analysis set was performed.

Group comparisons of the primary outcome were made using ANCOVA, with PA level at 3 months as dependent variable and group, baseline PA level, age and sex as independent variables. Age was entered into the model using restricted cubic splines with three knots, placed at the 10th, 50th and 90th percentile of the age distribution to account for non-linear relationship. In accordance with CONSORT, unadjusted models were fitted as sensitivity analysis. Assumptions of normally distributed and homoscedastic residuals were examined from graphs.

The secondary outcomes, self-reported PA and CAT were analysed using the same ANCOVA model as for the primary outcome. The mMRC and CRQ-SA were analysed using ordinal proportional odds models with 3-month measurements as dependent variable and group, baseline PA, age and sex as independent variables. Assumptions of proportional odds were examined by investigating the stability of the odds ratio between group, as estimated from binary logistic regression when dichotomising the scales at different locations.

The primary analysis approach was intention-to-treat (ITT). Analyses were performed on data with multiple imputation by chained equations (MICE) using partial mean matching to address potential bias from missing values due to dropout. The results were obtained by fitting the statistical model on each of the 30 imputed data sets and thereafter pooling the results using Rubin’s rule. The imputations were performed separately within the intervention arms. Additional details on the imputation model can be found in the online supplementary material (Supplementary Methods). Further, a planned complete case analysis was performed, i.e., including participants with full outcome measurements independent on adherence to the intervention. In accordance with the study protocol, a complementary per-protocol analysis was also planned with a predetermined definition of per-protocol population^24^. However, as only two participants from the ITT population did not adhere to per-protocol definition, resulting in close to identical results as the ITT analysis, the per-protocol analysis was deemed redundant.

A significance level of 5% was used in all statistical tests. The descriptive analyses were performed using SPSS (V.28.0; IBM Corp.), while the statistical modelling was performed using R v 4.3.0. The proportional odds models were fitted using the function *orm* from the R package *rms*^33^. The multiple imputations were made using the *mice* package^34^.

## Results

**Fig. 2:**
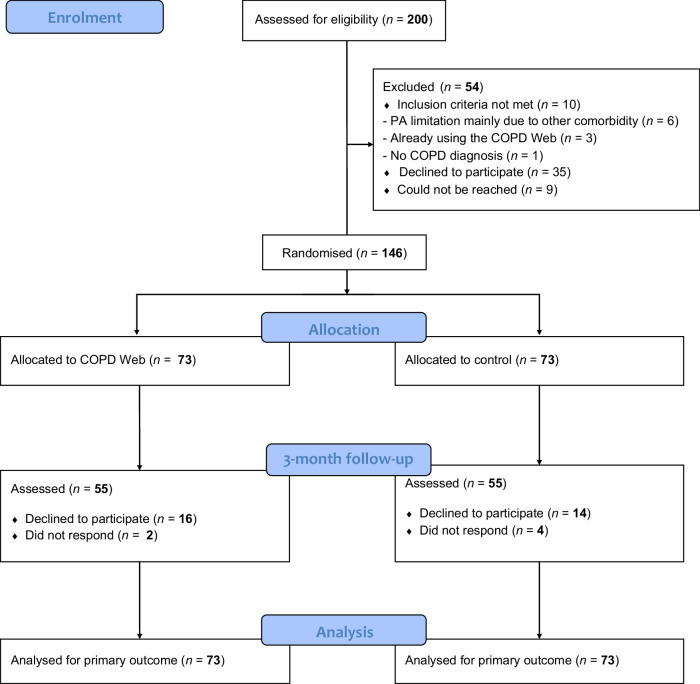
Flowchart of the participants in the RCT.

Of the 200 eligible participants, 146 were randomised to either the intervention (*n* = 73) or control (*n* = 73) group and included in the analysis (Fig. 2). Baseline characteristics of participants are shown in Table 1. Participants who had missing data on PA at 3 months had a significantly higher CAT-score (*p* = 0.04) and took fewer steps per day (*p* < 0.01) at baseline than those who had full outcome measurements (see results from the dropout analysis in Supplementary Table 1).

**Table 1:** Participant characteristics.

Characteristics	Total *n* = 146	COPD Web *n* = 73	Control *n* = 73
Age (years)	69.5 ± 6.7	68.8 ± 7.0	70.1 ± 6.4
Sex: female/male	69/77	37/36	32/41
BMI	26.4 ± 5.0	25.9 ± 4.6	27.0 ± 5.5
FEV_1,_ % of predicted (*n* = 142 (70/72))	60.7 ± 19.1	62.6 ± 19.1	58.8 ± 19.1
FEV_1_/FVC (%) (*n* = 143 (72/71))	55.2 ± 11.5	55.9 ± 10.2	54.5 ± 12.7
Daily steps^a^ (*n* = 143)	6082 ± 3663	6135 ± 3567	6030 ± 3780
Daily step of all valid days^b^ (*n* = 143)	5917 ± 3508	6070 ± 3471	5766 ± 3562

Values are mean ± SD or *n*.

*FEV*_*1*_ predicted forced expiratory volume in 1 s, *FEV*_*1*_*/FVC* ratio between FEV1 and forced vital capacity (FVC).

^a^Measurements of ≥4 valid weekdays with at least 8 h of daytime wearing time.

^b^For comparison between studies according to the international task force on physical activity^28^.

### Primary outcome

**Fig. 3:**
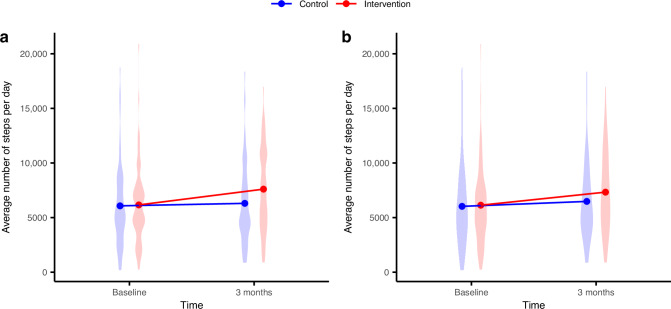
Mean daily steps and distribution for groups at baseline and at 3-month follow-up. **a** Intention-to-treat analysis. **b** Complete case analysis.

No significant difference in change in daily steps between the groups was seen at 3 months in the ITT analysis, 1295 steps (95% CI: [−365, 2955], *p* = 0.12). The complete case analysis (*n* = 98) showed, however, a significant and clinically relevant difference of 1491 steps per day (95% CI: [374, 2607], *p* = 0.01) in favour of the intervention group. Figure 3 shows the distribution of PA at baseline and 3 months for the groups in the ITT (Fig. 3a) and the complete case analysis (Fig. 3b), respectively.

### Secondary outcomes

Self-assessed PA level was significantly higher in the intervention group compared to control group in both the ITT 1.8 (95% CI: [0.3, 3.4], *p* = 0.02) and the complete case 1.8 (95% CI: [0.5, 3.0], *p* = 0.01) analysis. No other between-group differences were found in the secondary outcomes. Results of the ITT analyses are presented in Table 2 while the complete case analyses are provided as online supplementary material (Supplementary Table 2).

**Table 2:** Secondary outcomes—intention-to-treat analysis.

	COPD Web			Control			Between-group difference (95% CI)
	Baseline	3 months	Within-group difference	Baseline	3 months	Within-group difference	
Self-assessed PA level (3-19)	9.3 ± 4.1	11.1 ± 4.1	1.7 ± 4.4	10.0 ± 4.1	9.6 ± 3.9	−0.4 ± 4.1	1.8 (0.3, 3.4, *p* = 0.02)
CAT (1–40)	12.7 ± 6.4	9.0 ± 7.7	−3.6 ± 7.0	13.8 ± 6.9	9.0 ± 7.3	−4.8 ± 8.8	0.6 (−1.7, 2.8, *p* = 0.61)
mMRC							
0	5 (6.8)	9.1 (12.6)		4 (5.5)	2 .6 (3.6)		OR: 1.00 (0.52, 2.76, *p* = 0.74)
1	37 (50.7)	34.3 (47.0)		32 (43.8)	35.3 (48.4)		
2	20 (27.4)	18.0 (24.6)		14 (19.2)	24.2 (33.2)		
3	8 (11.0)	7.4 (10.1)		15 (20.5)	8.2 (11.2)		
4	3 (4.1)	4.2 (5.7)		8 (11.0)	2.7 (3.7)		
CRQ:							
Dyspnoea	5.4 ± 1.4	5.3 ± 1.4	−0.1 ± 0.9	5.3 ± 1.3	5.4 ± 1.2	0.2 ± 1.3	OR: 0.60 (0.58, 2.16, *p* = 0.13)
Fatigue	4.3 ± 1.2	4.5 ± 1.4	0.2 ± 1.1	4.1 ± 1.2	4.5 ± 1.1	0.3 ± 1.3	OR: 0.89 (0.55, 2.37, *p* = 0.75)
Emotion	5.2 ± 1.0	5.4 ± 1.0	0.2 ± 0.8	4.9 ± 1.1	5.2 ± 0.9	0.3 ± 1.0	OR: 1.22 (0.55, 2.45, *p* = 0.61)
Mastery	5.8 ± 1.2	6.0 ± 1.2	0.2 ± 1.1	5.5 ± 2.5	5.8 ± 1.1	0.3 ± 2.3	OR: 1.01 (0.54, 2.49, *p* = 0.98)

Baseline and 3-month data are presented as mean ± SD or *n* (%). Within-group-group differences are means ± SD. Between-group differences are presented as mean or odds ratio with 95% CI. CAT: COPD assessment test, higher values indicate greater impact of COPD; CRQ-SA: self-administrated chronic respiratory questionnaire, higher values indicate better health; Self-assessed PA and exercise: indicator questions according to the Swedish National Board of Health and Welfare, values of ≥11 correspond to ≥150 min of at least moderate PA/week; *mMRC* modified Medical Research Council dyspnoea scale, higher values indicate more dyspnoea; *PA* physical activity.

Nine of the participants reported COPD-related healthcare contacts at 3 months, five in the intervention and four in the control group, of whom two of the controls had been hospitalised. Furthermore, nine participants in the intervention group and eight in the control group had participated in COPD-related education or in a PR programme after being enrolled to the study.

## Discussion

The ITT analysis did not confirm our hypothesis that use of web-based self-management support along with a pedometer would increase objectively measured PA level at 3 months compared to usual care combined with a pedometer in people with COPD. However, the web-based self-management support did increase self-reported PA level, and among those with complete step data at 3 months, a significant and clinically relevant larger increase in objectively measured PA level was found in favour of the intervention group. No between-group differences were seen in COPD-related symptoms or HRQOL which, at least partly, could be explained by low baseline symptom burden and high baseline HRQOL, thus reducing the room for improvement.

The positive effect on self-reported PA level among those who had access to the COPD Web was comparable to that found in the pilot trial^23^. The fact that self-reported and not objectively measured PA increased may reflect that PA is a complex outcome. Self-reported outcome measures may capture PA in a broader sense with focus on the PA experience rather than the actual performance, i.e., number of steps^28^. Some discrepancy between subjective and objective PA measurement could also be due to the risk of recall bias when PA is self-reported^35^. Despite the lack of a statistically significant increase in objective PA, the observed difference surpassed the MCID for PA in COPD, which ranges from 600 to 1100 steps per day^7^. Additionally, the increase in self-reported PA levels supports these findings and is noteworthy in its own right, as higher self-reported PA has been linked to key clinical outcomes, including reduced mortality in older adults^36^. Furthermore, among individuals with COPD, increased self-reported PA has been associated with improved clinical outcomes such as physical capacity, HRQoL and reduced dyspnoea^37^. Nevertheless, the result indicates that the intervention had a positive impact on the participants’ everyday activities. Regarding the effect on objectively measured PA, our results from the complete case analyses suggest that the COPD Web intervention may be effective in increasing PA in some individuals, while others may need more support or other type of interventions. This is in line with Troosters et al. ^38^, who suggest that interventions in people with COPD with low exercise tolerance or severe symptom burden should primarily focus on increasing exercise tolerance before enhancing PA. Notably, those who were lost at follow-up, thus included in the ITT but not the complete case analysis, had lower baseline PA level, compared to those who completed the study procedures. This indicates that less active individuals may need additional support, which is in accordance with a previous systematic review and meta-analysis demonstrating that individuals with higher compared to lower baseline PA level showed greater improvements in steps per day after an intervention^39^. The need for additional support to increase PA in subgroups of people with COPD is also supported by the information obtained from our qualitative interviews in a subset of the participants in the RCT with a wide variation in characteristics and of using the tool^40^. Within this subset, we observed that for those who were ready and capable to use the COPD Web, it seemed to provide practical, emotional, and psychological help to incorporate measures needed for a behaviour change resulting in increased PA levels. The qualitative interviews also revealed that the COPD Web seemed to strengthen or weaken the perception of control by either providing support or being too demanding on the user, supporting the notion that a web-based self-management intervention might be a suitable and effective alternative to improve PA in some, but not all people with COPD^40^. These data also suggest that a potentially explanatory mechanism for the results seen among those who completed the full 3-month intervention period is that the COPD Web was successful in empowering the participants to boost their level of agency and their ability to improve PA levels. Similarly, Slevin et al.^41^ reported that those who had the capacity to engage in an eHealth tool perceived that it supported their self-management ability, increased their self-efficacy to manage the disease, reduced their anxiety, and facilitated personalised care

Finding ways to promote a beneficial PA behaviour in people with COPD is indeed a challenge and the most optimal intervention is yet to be determined^8^. The present study provides further support for the notion that low PA levels can occur across a broad spectrum of people with COPD, not only among those with a high symptom and disease burden. Previous interventions that enhanced short-term PA have, for instance, combined a pedometer with five individual counselling sessions^42^ or with a tele-coaching intervention including a motivational interview with a HCP^19^. Other studies had an online social support forum included in the eHealth tool^16,17^. The present study was designed to have minimal involvement from the HCPs as well as the researchers, even though it was a standalone intervention and not delivered following a PR programme. To facilitate PA, interactive parts, e.g., weekly step goal, step registration, and automated feedback, were included in the COPD Web, components that have shown to be beneficial for behavioural change^9^. Although our findings suggest that this was only sufficient to increase PA levels among those with complete outcome data, that is, those who were shown to have more preserved baseline PA. The intervention might have been more successful for those with a lower baseline PA if individualised support from the HCPs and/or the researchers had been included as part of the intervention. This could have boosted the participants’ self-efficacy in improving PA and guided them in finding an appropriate level of PA that could be incorporated into their daily lives, as suggested by Kosteli et al.^43^. Evidently, eHealth interventions may not be the best treatment option for all people with COPD but should, according to Kermelly et al.^44^, be incorporated into regular care as an adjunct or enhancement to existing self-management interventions. In clinical practice, it seems important for HCPs to have a patient-centric approach when considering the suitability of a digital self-management support alternative for their patients.

### Methodological considerations

Strengths of the study are that the COPD Web was developed in co-creation with end-users and had a pragmatic study design with participation at a distance with few restrictions on what other interventions the participants took part in, which reflects reality. Participation in other interventions, such as COPD-related education or in a PR programme, might be a confounding factor, but few participants had received such interventions, and the distribution was comparable between the groups at the 3-month follow-up. The pragmatic design also allowed the study to proceed despite the restrictions of the COVID-19 pandemic that designated people with COPD at high risk for severe symptoms^45^, although it is difficult to determine to what extent the pandemic affected the study results. Other strengths are that PA level was measured in accordance with the recommendations from the international task force on PA^28^ and that we used a robust imputation procedure to address the potential bias that can occur when values are missing due to dropout^46^. The HCP’s input, if any, was kept to a minimum to preserve the participant’s self-management and no participant was after the pandemic asked to seek their PHCs for updated spirometry or health data in case something was missing. On the other hand, this limits the possibility of determining the participant’s capacity and compliance with the intervention.

There are some limitations to address. The extent to which the COPD Web was used is not known since the new interpretation of GDPR during the study made user analyses prohibited. The dropout rate was greater than expected, which may partly be due to the pandemic. The main reasons for refusing participation in the follow-up assessment were e.g., that they had stopped their normal social activities or had stopped exercising, which indicated that they saw the follow-up as pointless. Another reason was that people with the opportunity left the cities and spent more time in their summer houses where they had limited mail and internet connection. It is also possible that the study design, which involved few personal contacts with the HCPs and the researchers, and the fact that the intervention was not personally tailored could have contributed to participants withdrawing from the study. It is worth noticing, though, that 67% of all participants had complete outcome data, despite the potential negative impact of the COVID-19 pandemic, and among those, a statistically significant but also clinically relevant mean improvement of more than 1400 steps per day was observed. Another limitation is that the health literacy and patient activation among the participants are not known. This means that we do not know if they had the skills and confidence required for a self-management intervention, as well as if they were motivated and had the capacity to take action^47^. Moreover, despite that the study evaluated an eHealth intervention, we did not consider digital health literacy when including participants in the study, which has been reported to be a common patient barrier to the adoption of eHealth interventions^15^. Lastly, we have not yet evaluated the 12-month effects of the intervention, which will show if the intervention had a long-term effect on PA level. As part of the previously mentioned pilot trial^23^, a qualitative study of participants who had access to the COPD Web for 12 months found that time was needed to be able to incorporate knowledge from the tool into everyday life, indicating that changes in health behaviour such as increasing PA level may take up to a year^22^.

In summary, the ITT analysis found that a web-based self-management support demanding minimal effort from HCPs, was not sufficient to increase PA level in people with COPD. Still, the complete case analysis found a significant and clinically relevant increase in level of PA, indicating a benefit of the web-based support in participants with complete step data. Participants in both control and intervention groups lost to follow-up had a lower PA level at baseline, which likely is one of the factors that contributed to the observed differences in the ITT and complete case analysis, and which highlights the need for additional support and interventions in those with lower baseline PA. It is important to identify people who may benefit from using web-based support for increasing their PA levels in order to use HCP resources in primary healthcare as effectively as possible.

## Supplementary information

Supplemental Material

Nr-reporting-summary

## Data Availability

Data are available upon reasonable request.
