# Supplementary material for: Short-term effects on physical activity level with web-based self-management support in people with COPD: a randomised controlled trial
Source: NPJ Prim Care Respir Med. 2024 Oct 24;34:32. doi: 10.1038/s41533-024-00394-7 (PMC11502778; doi:10.1038/s41533-024-00394-7)
Supplement: Supplementary file 1 — Supplemental Material [file 41533_2024_394_MOESM1_ESM.pdf]

Supplementary Methods

Information on imputation

Multiple imputations were performed by group using Multiple Imputation by Chained Equations (MICE). For the intervention group and the control group separately, 30 imputed data sets each were generated, using partial mean matching. The following variables were included in the imputation model: sex, bmi, age, self-assessed physical level (PA) at baseline and 3-months, PA level at baseline and at 3 months, CAT at baseline and 3-months, modified Medical Research Council dyspnea scale (mMRC) at baseline and 3-months, % predicted FEV_1 at baseline, each of the four dimension of the Chronic Respiratory Questionnaire (Dyspnea, Fatigue, Emotion and Mastery) at baseline and 3-months, “Bas_1” and “Bas_2” . Variable Bas_1 answered if the participant at baseline had sought healthcare for its COPD the past 12-months and BAS_2 the number of healthcare contacts the past 12-months.

R -code for generating the imputations is given below:

```
library(mice)

library(tidyverse)

df_intervention <- df %>%

  filter(Intervention == "Intervention") %>%

  select(Sex,

       BMI,

       Age,

       Bas_Avg_Steps_Weekdays,

       Month3_Avg_Steps_Weekdays,

       contains("Soc_Ind"),

       contains("EQ5D"),

       FEV1_pred,

       Bas_mMRC,

       contains("CAT"),

       contains("mMRC"),

       contains("CRQ"),

       Bas_1,

       Bas_2

       )


df_control <- df %>%

  filter(Intervention == "Control") %>%

  select(Sex,
```

```
        BMI,
        Age,
        Bas_Avg_Steps_Weekdays,
        Month3_Avg_Steps_Weekdays,
        contains("Soc_Ind"),
        contains("EQ5D"),
        FEV1_pred,
        Bas_mMRC,
        contains("CAT"),
        contains("mMRC"),
        contains("CRQ"),
        Bas_1,
        Bas_2
        )
)
Seed <- TeachingDemos::char2seed("Kolwebben")
imp_int <- mice(df_intervention,
            seed = Seed,
            m = 30,
            method = "pmm",
            maxit = 20,
            printFlag = FALSE)
imp_con <- mice(df_control,
            seed = Seed,
            m = 30,
            method = "pmm",
            maxit = 20,
            printFlag = FALSE)

int_long <- complete(imp_int, action = "long", include = TRUE) %>%
  mutate(Intervention = "Intervention")
con_long <- complete(imp_con, action = "long", include = TRUE)%>%
  mutate(Intervention = "Control")

imp_df <- int_long %>%
```

```
bind_rows(con_long) %>%
as.mids(.id = "Code")
```

Supplementary Tables

Table 1a. Drop out analysis. P-values calculated from t-test for continuous variables, and chi-square test for mMRC

	**Completed follow-up (n=98)**	**Missing at follow-up (n=48)**	**P-value**
Baseline PA	6538 ±3307	5149 ±4185	<0.01
Age (years)	69.9 ±6.1	68.6 ±7.8	0.52
BMI	26.4 ±4.9	26.5 ±5.6	0.79
Males	51 (52%)	26 (54%)	0.81
%predicted FEV_1	62 ±18	58 ±22	0.46
CAT (1–40)	12 ±6	15 ±7	0.04
mMRC			0.18
0	6 (6.1%)	3 (6.3%)	
1	50 (51%)	19 (40%)	
2	25 (26%)	9 (19%)	
3	11 (11%)	12 (25%)	
4	6 (6.1%)	5 (10%)	
CRQ:			
Dyspnea	5.5 ±1.3	5.0 ±1.3	0.02
Fatigue	4.4 ±1.2	3.9 ±1.1	0.02
Emotion	5.1 ±1.0	4.8 ±1.1	0.10
Mastery	5.7 ±1.3	5.5 ±2.9	0.01

Data are presented as mean ± SD or n (%).

CAT: COPD assessment test, higher values indicate greater impact of COPD; CRQ: self-administrated chronic respiratory questionnaire, higher values indicate better health; mMRC: modified Medical Research Council dyspnea scale, higher values indicate more dyspnea; PA: physical activity

Table 1b. Drop out analysis comparing baseline data for participants with and without completed follow-up within groups. P-values calculated from t-test for continuous variables, and chi-square test for mMRC

	Control group			Intervention group		
	Completed follow-up (n = 48)	Missing at follow-up (n = 25)	P-value	Completed follow-up (n = 50)	Missing at follow-up (n = 23)	P-value
Baseline PA	6981 ± 3779	4241 ± 3130	<0.01	6114 ± 2753	6182 ± 5007	0.31
Age	70.1 ±6.4	70.0 ±6.7	0.94	69.7 ±5.9	67.0 ±8.7	0.40
BMI	26.9 ±5.4	27.2 ±5.9	0.75	25.9 ±4.3	25.8 ±5.3	0.48
Males	26 (54%)	15 (60%)	0.64	25 (50%)	11 (48%)	0.86
% predicted FEV_1	62 ±17	51 ±21	0.02	61 ±18	66 ±21	0.23
CAT (1–40)	12 ±6	15 ±7	0.07	12 ±6	15 ±7	0.31
mMRC			0.02			0.44
0	2 (4.3%)	2 (8.0%)		4 (8.2%)	1 (4.3%)	
1	23 (49%)	8 (32%)		26 (53%)	11 (48%)	
2	10 (21%)	4 (16%)		14 (29%)	5 (22%)	
3	7 (15%)	8 (32%)		4 (8.2%)	4 (17%)	
4	5 (11%)	3 (12%)		1 (2.0%)	2 (8.7%)	
CRQ:						
Dyspnea	5.4 ±1.2	4.9 ±1.5	0.08	5.5 ±1.4	5.1 ±1.2	0.14
Fatigue	4.3 ±1.2	3.8 ±1.2	0.07	4.5 ±1.2	4.1 ±1.1	0.18
Emotion	5.0 ±1.0	4.7 ±1.2	0.46	5.3 ±1.0	4.9 ±1.0	0.11
Mastery	5.6 ±1.4	5.5 ±3.9	0.13	5.9 ±1.1	5.6 ±1.3	0.52

Data are presented as mean ± SD or n (%).

CAT: COPD assessment test, higher values indicate greater impact of COPD; CRQ: self-administrated chronic respiratory questionnaire, higher values indicate better health; mMRC: modified Medical Research Council dyspnea scale, higher values indicate more dyspnea; PA: physical activity

Table 2. Secondary outcomes – complete case analysis

	COPD Web			Control			
	Baseline	3 months	Within-group difference	Baseline	3months	Within-group difference	Between-group difference
Self-assessed PA level (3-19)	9.3 ± 4.1	10.6 ± 3.9	1.3 ± 3.6	10.0 ± 4.1	9.6 ± 3.8	-1.0 ± 3.5	1.8 (0.5, 3.0, p = 0.01)
CAT (1–40)	12.7 ± 6.4	12.0 ± 6.6	-0.4 ± 3.3	14.0 ± 6.8	12.0 ± 5.9	-1.1 ± 3.9	0.6 (-1.7, 2.8, p = 0.61)
mMRC							
0	5 (6.8)	4 (5.5)		4 (5.5)	2 (2.7)		OR:1.00 (0.58, 2.77, p = 0.61)
1	37 (50.7)	27 (37.0)		32 (43.8)	26 (35.6)		
2	20 (27.4)	14 (19.2)		14 (19.2)	19 (26.0)		
3	8 (11.0)	6 (8.2)		15 (20.5)	6 (8.2)		
4	3 (4.1)	4 (5.5)		8 (11.0)	2 (2.7)		
CRQ:							
Dyspnea	5.4 ± 1.4	5.3 ± 1.5	-0.1± 0.5	5.3 ± 1.3	5.5 ± 1.2	0.1± 1.0	OR: 0.55 (0.58, 2.17, p = 0.07)
Fatigue	4.3 ± 1.2	4.6 ± 1.4	0.1 ± 0.8	4.1 ± 1.2	4.4 ± 1.1	0.1 ± 1.1	OR: 1.03 (0.58, 2.17, p = 0.92)
Emotion	5.2 ± 1.0	5.4 ± 1.0	0.0 ± 0.6	4.9 ± 1.1	5.1 ± 0.8	0.1 ± 0.8	OR: 1.31 (0.57, 2.20, p = 0.43)
Mastery	5.8 ± 1.2	5.9 ± 1.3	0.0 ± 0.8	5.5 ± 2.5	5.8 ± 1.1	0.3 ± 1.0	OR: 0.89 (0.57, 2.27, p = 0.74)

Baseline and 3-month data are presented as mean ± SD or n (%). Within-group-group differences are means ± SD. Between-group differences are presented as mean or odds ratio with 95% CI.

CAT: COPD assessment test, higher values indicate greater impact of COPD; CRQ-SA: self-administrated chronic respiratory questionnaire, higher values indicate better health; Self-assessed PA and exercise: indicator questions according to the Swedish National Board of Health and Welfare, values of ≥ 11 correspond to ≥ 150 min of at least moderate PA/week; mMRC: modified Medical Research Council dyspnea scale, higher values indicate more dyspnea; PA: physical activity
