# Supplementary material for: Short-term effects on physical activity level with web-based self-management support in people with COPD: a randomised controlled trial
Source: NPJ Prim Care Respir Med. 2024 Oct 24;34:32. doi: 10.1038/s41533-024-00394-7 (PMC11502778; doi:10.1038/s41533-024-00394-7)
Supplement: Supplementary file 2 — Nr-reporting-summary [file 41533_2024_394_MOESM2_ESM.pdf]

nature portfolio

Corresponding author(s): NPJPCRM-01880

Last updated by author(s): Aug 28, 2024

Reporting Summary

Nature Portfolio wishes to improve the reproducibility of the work that we publish. This form provides structure for consistency and transparency in reporting. For further information on Nature Portfolio policies, see our Editorial Policies and the Editorial Policy Checklist.

Statistics

For all statistical analyses, confirm that the following items are present in the figure legend, table legend, main text, or Methods section.

n/a	Confirmed	
☐	☒	The exact sample size (*n*) for each experimental group/condition, given as a discrete number and unit of measurement
☐	☒	A statement on whether measurements were taken from distinct samples or whether the same sample was measured repeatedly
☐	☒	The statistical test(s) used AND whether they are one- or two-sided<br>*Only common tests should be described solely by name; describe more complex techniques in the Methods section.*
☐	☒	A description of all covariates tested
☐	☒	A description of any assumptions or corrections, such as tests of normality and adjustment for multiple comparisons
☐	☒	A full description of the statistical parameters including central tendency (e.g. means) or other basic estimates (e.g. regression coefficient) AND variation (e.g. standard deviation) or associated estimates of uncertainty (e.g. confidence intervals)
☐	☒	For null hypothesis testing, the test statistic (e.g. *F*, *t*, *r*) with confidence intervals, effect sizes, degrees of freedom and *P* value noted<br>*Give P values as exact values whenever suitable.*
☒	☐	For Bayesian analysis, information on the choice of priors and Markov chain Monte Carlo settings
☒	☐	For hierarchical and complex designs, identification of the appropriate level for tests and full reporting of outcomes
☒	☐	Estimates of effect sizes (e.g. Cohen's *d*, Pearson's *r*), indicating how they were calculated

Our web collection on statistics for biologists contains articles on many of the points above.

Software and code

Policy information about availability of computer code

Data collection	Level of PA will be objectively measured seven consecutive days using an accelerometer (DynaPort, McRoberts BV, the Netherlands).
Data analysis	The descriptive analyses were performed using SPSS (V.28.0; IBM Corp.), while the statistical modelling was performed using R v 4.3.0. The proportional odds models were fitted using the function orm from the R package rms . The multiple imputations were made using the mice package.<br>Harrel, F.E.J. _rms: Regression Modeling Strategies_. r package version 6.7-0. (2023).<br>van Buuren, S. & Groothuis-Oudshoorn, K. mice: Multivariate Imputation by Chained Equations in R. Journal of Statistical Software 45, 1 - 67 (2011).

For manuscripts utilizing custom algorithms or software that are central to the research but not yet described in published literature, software must be made available to editors and reviewers. We strongly encourage code deposition in a community repository (e.g. GitHub). See the Nature Portfolio guidelines for submitting code & software for further information.

Data

Policy information about availability of data

All manuscripts must include a data availability statement. This statement should provide the following information, where applicable:

- Accession codes, unique identifiers, or web links for publicly available datasets
- A description of any restrictions on data availability
- For clinical datasets or third party data, please ensure that the statement adheres to our policy

Data are available upon reasonable request.

Research involving human participants, their data, or biological material

Policy information about studies with human participants or human data. See also policy information about sex, gender (identity/presentation), and sexual orientation and race, ethnicity and racism.

Reporting on sex and gender	Sex is reported as participant characteristics. No subgroup analyses due to sex were included in the protocol nor perfomed in the final analysis.
Reporting on race, ethnicity, or other socially relevant groupings	No race, ethnicity or other socially relevant groupings were collected. Participants with stable COPD were eligible for inclusion if they (1) could read and understand Swedish, (2) had a smartphone, tablet or computer with access to internet, (3) did not have dementia or other psychiatric condition that may prevent understanding of the intervention, (4) did not have severe comorbidity that could be considered as the contributing factor for limitation in PA, and (5) did not already use the COPD Web. No race. ethnicity or other socially relevent groupings were collected.
Population characteristics	Age, Sex, BMI, FEV1 % predicted, FEV1/FVC (%) were collected participant characteristics besides the primary and secondary outcomes. The primary outcome was the difference in PA level, measured as steps per day. Secondary outcomes were self-reported PA level measured by indicator questions from the National Board of Health and Welfare in Sweden, dyspnea measured by modified Medical Research Council dyspnea scale (mMRC), HRQOL measured by the self-administered Chronic Respiratory Questionnaire (CRQ-SA), COPD-related symptoms measured by the COPD Assessment Test (CAT), and self-reported healthcare contacts. The characteristic mainly describes the severity of COPD for the participants included in the study.
Recruitment	Participants were recruited through healthcare personels at 25 primary healthcare units situated in urban and rural areas in 6 different County Councils in Sweden and via advertisements in newspapers and Facebook during the period of November 2018 to March 2021.
Ethics oversight	Ethical approval was received from the Regional Ethical Review Board in Umeå, Sweden (Dnr: 2018-274-31, 2019-05572).

Note that full information on the approval of the study protocol must also be provided in the manuscript.

Field-specific reporting

Please select the one below that is the best fit for your research. If you are not sure, read the appropriate sections before making your selection.

☒ Life sciences ☐ Behavioural & social sciences ☐ Ecological, evolutionary & environmental sciences

For a reference copy of the document with all sections, see nature.com/documents/nr-reporting-summary-flat.pdf

Life sciences study design

All studies must disclose on these points even when the disclosure is negative.

Sample size	The trial was designed to detect a mean difference of 1131 steps, which has previously been determined as a minimal important difference in people with COPD 7. Demeyer et al. 7 reported a standard deviation in PA level of 2193 steps after 3 months of Pulmonary Rehabilitation (PR) in a sample of people with COPD. Assuming the same standard deviation in the present trial and a dropout rate of 20% in both groups, 144 participants would be required to reach a power of 80% for detecting a group difference of 1131 steps when using a two-tailed independent sample t-test.
Data exclusions	Full details on outcomes and assessment procedures are available in the study protocol. According to recommendations only measurements with ≥ 4 valid weekdays were included i.e., weekends were excluded as well as weekdays with < 8 hours of daytime accelerometer wearing time.
Replication	The study was designed as a pragmatic trial. The design aimed to minimise the effort from healthcare professionals and increase the possibility of self-management for people with COPD with a minimum of restrictions besides the inclusioncriteria (1) could read and understand Swedish, (2) had a smartphone, tablet or computer with access to internet, (3) did not have dementia or other psychiatric condition that may prevent understanding of the intervention, (4) did not have severe comorbidity that could be considered as the contributing factor for limitation in PA, and (5) did not already use the COPD Web.

Randomization	Participants were randomly allocated in a 1:1 allocation ratio to usual care with or without access to the COPD Web, after completion of the baseline assessment.
Blinding	Blinding of participants and outcome assessors was not applicable since data was either self-reported or objectively measured by an accelerometer.

Reporting for specific materials, systems and methods

We require information from authors about some types of materials, experimental systems and methods used in many studies. Here, indicate whether each material, system or method listed is relevant to your study. If you are not sure if a list item applies to your research, read the appropriate section before selecting a response.

Materials & experimental systems

n/a	Involved in the study	
☒	☐	Antibodies
☒	☐	Eukaryotic cell lines
☒	☐	Palaeontology and archaeology
☒	☐	Animals and other organisms
☐	☒	Clinical data
☒	☐	Dual use research of concern
☒	☐	Plants

Methods

n/a	Involved in the study	
☒	☐	ChIP-seq
☒	☐	Flow cytometry
☒	☐	MRI-based neuroimaging

Clinical data

Policy information about clinical studies

All manuscripts should comply with the ICMJE guidelines for publication of clinical research and a completed CONSORT checklist must be included with all submissions.

Clinical trial registration	ClinicalTrials.gov (NCT03746873)
Study protocol	Stenlund, T., Nyberg, A., Lundell, S. & Wadell, K. Web-based support for self-management strategies versus usual care for people with COPD in primary healthcare: a protocol for a randomised, 12-month, parallel-group pragmatic trial. BMJ Open 9, e030788 (2019).
Data collection	Participants were recruited through healthcare profesionals at 25 primary healthcare units situated in urban and rural areas in 6 different County Councils in Sweden and via advertisements in newspapers and Facebook during the period of November 2018 to March 2021. The last follow-up assessments were finalized in June 2022. Its a pragmatic, parallel-group RCT in a primary healthcare context with data from baseline and a 3- and 12-month follow-up, of which the present study will report on the 3-month results. Data was collected via questionnaires and accelerometers worn for 7 consecutive days. The material was sent by mail to the home of the participant who, after wearing the accelerometer for the predetermined 7 days and answering the questions, sent in the material.
Outcomes	The primary outcome was the difference in PA level, measured as steps per day, between intervention and control groups at 3 months. The PA level was objectively measured seven consecutive days using an accelerometer (DynaPort, McRoberts BV, the Netherlands). According to recommendations only measurements with ≥ 4 valid weekdays were included i.e., weekends were excluded as well as weekdays with < 8 hours of daytime accelerometer wearing time . Secondary outcomes were self-reported PA level measured by indicator questions from the National Board of Health and Welfare in Sweden, dyspnea measured by modified Medical Research Council dyspnea scale (mMRC), HRQOL measured by the self-administered Chronic Respiratory Questionnaire (CRQ-SA), COPD-related symptoms measured by the COPD Assessment Test (CAT), and self-reported healthcare contacts.

Plants

Seed stocks	*Report on the source of all seed stocks or other plant material used. If applicable, state the seed stock centre and catalogue number. If plant specimens were collected from the field, describe the collection location, date and sampling procedures.*
Novel plant genotypes	*Describe the methods by which all novel plant genotypes were produced. This includes those generated by transgenic approaches, gene editing, chemical/radiation-based mutagenesis and hybridization. For transgenic lines, describe the transformation method, the number of independent lines analyzed and the generation upon which experiments were performed. For gene-edited lines, describe the editor used, the endogenous sequence targeted for editing, the targeting guide RNA sequence (if applicable) and how the editor was applied.*
Authentication	*Describe any authentication procedures for each seed stock used or novel genotype generated. Describe any experiments used to assess the effect of a mutation and, where applicable, how potential secondary effects (e.g. second site T-DNA insertions, mosiacism, off-target gene editing) were examined.*
